# Phosphodiesterase 3A: a new player in development of interstitial cells of Cajal and a prospective target in gastrointestinal stromal tumors (GIST)

**DOI:** 10.18632/oncotarget.17010

**Published:** 2017-04-10

**Authors:** Pierre Vandenberghe, Perrine Hagué, Steven C. Hockman, Vincent C. Manganiello, Pieter Demetter, Christophe Erneux, Jean-Marie Vanderwinden

**Affiliations:** ^1^ Laboratory of Neurophysiology, Faculty of Medicine, Université Libre de Bruxelles, Brussels, Belgium; ^2^ Cardiovascular and Pulmonary Branch, National Heart, Lung, and Blood Institute, National Institutes of Health, Bethesda, MD, USA; ^3^ Department of Pathology, Erasme University Hospital, Université Libre de Bruxelles, Brussels, Belgium; ^4^ IRIBHM, Faculty of Medicine, Université Libre de Bruxelles, Brussels, Belgium

**Keywords:** transgenic mice, cilostazol, tissue array, KIT, cancer

## Abstract

We previously identified phosphodiesterase 3A (PDE3A) as a marker for interstitial cells of Cajal (ICC) in adult mouse gut. However, PDE3A expression and function during gut development and in ICC-derived gastrointestinal stromal tumors (GIST) remained unknown. Here we found that PDE3A was expressed throughout ICC development and that ICC density was halved in PDE3A-deficient mice. In the human imatinib-sensitive GIST882 cell line, the PDE3 inhibitor cilostazol halved cell viability (IC_50_ 0.35 μM) and this effect synergized with imatinib (Chou-Talalay's CI_50_ 0.15). Recently the compound 6-(4-(diethylamino)-3-nitrophenyl)-5-methyl-4,5-dihydropyridazin-3(2H)-one, or DNMDP was found to be cytotoxic selectively for cells expressing both PDE3A and Schlafen12 (SLFN12) (de Waal L et al. Nat Chem Bio 2016), identifying a new, non-catalytic, role for PDE3A. 108 out of 117 (92%) of our human GIST samples displayed both PDE3A and SLFN12 immunoreactivity. GIST882 cells express both PDE3A and SLFN12 and DNMDP decreased their viability by 90%. Our results suggest a role for PDE3A during ICC development and open novel perspectives for PDE3A in targeted GIST therapy, on one hand by the synergism between imatinib and cilostazol, a PDE3 inhibitor already in clinical use for other indications, and, on the other hand, by the neomorphic, druggable, PDE3A-SLFN12 cytotoxic interplay.

## INTRODUCTION

Gastrointestinal stromal tumors (GIST) are the most common sarcoma of the gastrointestinal tract. Approximately 85% of GIST harbor some somatic oncogenic (gain-of function) mutation of the tyrosine kinase receptor (RTK) KIT (a.k.a. CD117 or c-kit) [1]. STI-571 (imatinib mesylate, Gleevec™), the first tyrosine kinase inhibitor targeting KIT to be used in the clinics, epitomizes the targeted therapy approach. It remains nowadays the first-line medication for GIST patients, although most patients will ultimately progress under treatment [2]. Despite subsequent introduction of novel selective KIT inhibitors [3], [4], primary resistance and escapes during treatment remain major concerns in GIST therapy [5]. Therefore, studies of the signaling pathways downstream of the KIT RTK and its oncogenic mutant forms remain essential to refine GIST targeted therapy.

GIST derives from the mesenchymal interstitial cells of Cajal (ICC) - or their precursors - which form extensive networks within the muscularis propria of the gastrointestinal tract. ICC regulate gut motility, acting as pacemaker of the slow waves of depolarization of the smooth muscle layers and as mediators of neurotransmitter inputs from the enteric nervous system [6], [7]. KIT RTK is expressed in ICC throughout development and in postnatal life. KIT signaling is essential for ICC development and maintenance [8], [9]. KIT immunoreactivity (-ir) is widely accepted as a marker for ICC [10] and is also present in the majority of GIST [1].

We previously identified genes differentially expressed [11] in the KIT+ cell hyperplasia of a transgenic mouse model harboring Kit^K641E^ oncogenic mutant [12]. Those genes were identified by microarray in total antrum and validated by qPCR and immunostaining. One of the upregulated genes was the cyclic nucleotide phosphodiesterase 3A (PDE3A), which belongs to the large family of 11 cyclic nucleotide phosphodiesterases (PDE) [13]. PDE downregulate levels of cyclic adenosine monophosphate (cAMP) and cyclic guanosine monophosphate (cGMP) and thus control important molecular mechanisms such as cell proliferation, migration or apoptosis [14].

Expression of PDE3A has been reported in various tissues such as heart, brain, platelets, oocyte and PDE3A functions in cardiovascular and reproductive organs have been largely investigated [15]. Conversely, in the gut, although PDE3A-ir has been reported as marker for the KIT-ir ICC [16], the potential role of PDE3A in the biology of the KIT+ ICC or in the ICC-derived GIST remains unknown. Consequently, PDE3A deserves further consideration to better understand ICC physiology and, with PDE3A inhibitors already in clinical use for other indications [15], PDE3A could be a novel putative therapeutic target in GIST.

While the catalytic function of PDE3A (i.e. the regulation of cyclic nucleotides level) has attracted most attention, PDE3A was also found to interact with the scaffold proteins 14-3-3 and with the protein phosphatase 2 (PP2A) in HeLa cells [17]. Recently, a novel, non-catalytic, function for PDE3A was identified by predictive chemogenomics [18]. That extensive survey of hundreds of human cell lines identified cancer-cytotoxic modulators of PDE3A, epitomized by the DNMDP compound (6-(4-(diethylamino)-3-nitrophenyl)-5-methyl-4,5-dihydropyridazin-3(2H)-one). DNMDP turned out to be cytotoxic only for cells expressing both PDE3A and a protein named Schlafen 12 (SLFN12). DNMDP appeared to promote a neomorphic cytotoxic interaction between PDE3A and SLFN12, paving the way for a novel class of potential cancer therapeutic agents.

In this study, we firstly focused on the PDE3A expression pattern during mouse gut development, and analyzed the ICC phenotype in the PDE3A^-/-^ transgenic mouse model [19] to unravel the importance of PDE3A in ICC biology. Next, we studied the biochemical role of PDE3A *in vitro*, using the human GIST882 cell line, homozygous for the KIT^K642E^ oncogenic mutation and assessed by immunohistochemistry expression of PDE3A and SLFN12 in human GIST tissue arrays.

## RESULTS

### Characterization of PDE3A antibodies used for immunohistochemistry in ICC

The identification of ICC in tissues relies on selective immunomarkers epitomized by KIT-ir. We previously reported [16] that KIT-ir ICC in human and mouse gut were selectively labelled by a PDE3A antibody (Ab) raised in sheep against a peptide sequence of human PDE3A (hPDE3A 1095-1110) [20]. As mouse and human peptide sequences differ by five amino acids over a total of 16 amino acids in the immunogenic peptide (Supplementary Figure 1), a second antibody, raised in rabbit against mouse PDE3A (mPDE3A 1098-1115) [21], was used in comparison.

**Figure 1:**
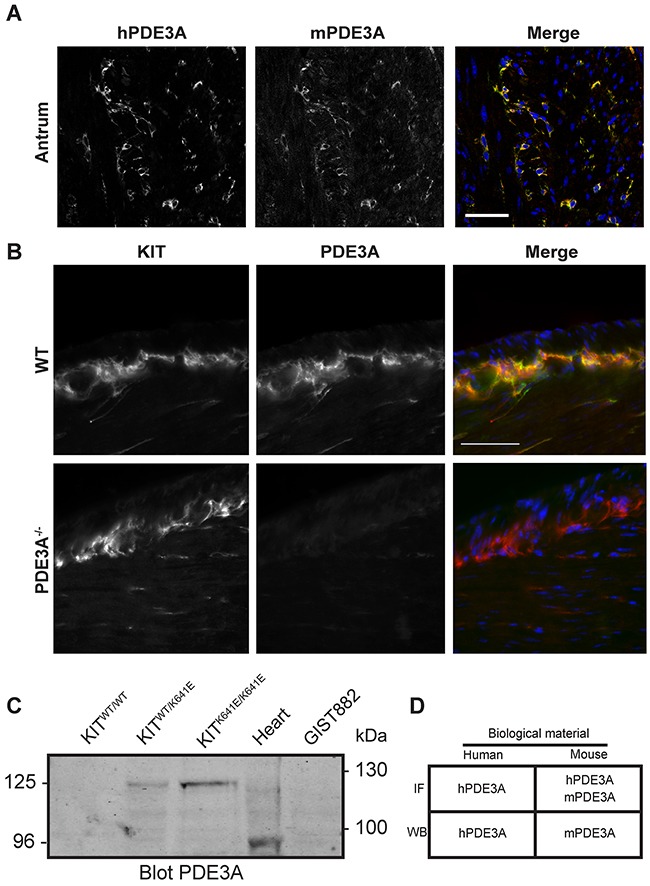
Validation of PDE3A antibodies in mouse antrum **(A)** Immunofluorescence. Left panel: antibody against human PDE3A (hPDE3A). Middle panel: antibody against mouse PDE3A (mPDE3A). Right panel: merged images of hPDE3A in green and mPDE3A in red. Immunoreactivity (-ir) for the two antibodies colocalized in ICC of adult mouse antrum. Confocal microscopy. Scale bar = 50μm. **(B)** Immunofluorescence. Left panel: KIT-ir in WT and PDE3A^-/-^ adult mice antrum. Middle panel: hPDE3A-ir in the two genotypes. Right panel: merged images of KIT-ir in red and hPDE3A-ir in green. hPDE3A-ir colocalized with KIT-ir in WT antrum while no hPDE3A-ir was detectable in PDE3A^-/-^ antrum. Widefield microscopy. Scale bar = 50μm. **(C)** Western blot probed with mPDE3A antibody showed bands around 125 kDa in Kit^WT/K641E^ and KitK^641E/K641E^ mouse antrum and around 95 kDa in mouse heart. mPDE3A antibody did not detect any band in Kit^WT/WT^ sample nor in human GIST882 cell line. 50μg protein/lane. **(D)** Summary of hPDE3A and mPDE3A immunoreactivity by immunofluorescence (IF) and western blot (WB) of human and mouse samples.

Double IF showed that the signals for hPDE3A Ab and mPDE3A Ab colocalized in the same interstitial cells in the muscularis propria of the mouse gut, indicating that the hPDE3A antibody was appropriate to recognize mouse PDE3A (Figure 1A).

Next, we performed double IF using the hPDE3A Ab and a KIT Ab as ICC marker on WT and PDE3A^-/-^ mouse antrum. PDE3A-ir was detected selectively in the KIT-ir ICC in WT antrum while no PDE3A-ir was detectable in PDE3A^-/-^ antrum, confirming the PDE3A specificity of the hPDE3A antibody (Figure 1B). PDE3A expression in the mouse antrum was also assessed by Western blot using the mPDE3A Ab (Figure 1C). As ICC represent only a tiny percentage of the total cell population in the antrum wall [16], total lysates of antrum of Kit^WT/WT^, Kit^WT/K641E^ and Kit^K641E/K641E^ mice harboring the Kit^K641E^ oncogenic Kit mutation leading to ICC hyperplasia were probed. No signal was detectable in Kit^WT/WT^ while a ~125 kDa band was detected in Kit^WT/K641E^ and Kit^K641E/K641E^ total protein extracts. Total mouse heart lysate, used as positive control, showed a ~96 kDa band [19]. Noteworthy, no signal was detected in GIST882 human cell line extract, indicating that mPDE3A Ab did not recognize human PDE3A protein by Western blotting.

In summary (Figure 1D), both hPDE3A and mPDE3A Abs were found suitable for the detection of PDE3A by IF in mouse gut tissues, while for Western blotting, hPDE3A Ab and mPDE3A Ab worked exclusively on human or mouse extracts, respectively.

### PDE3A is developmentally regulated in mouse antrum ICC

**Figure 2:**
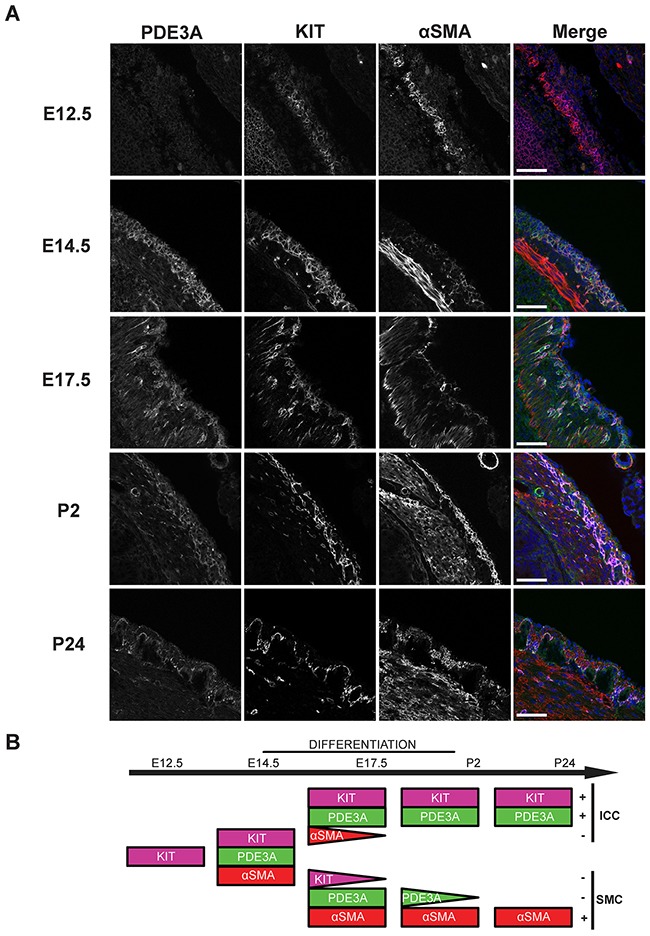
PDE3A expression is developmentally regulated during mouse gut embryogenesis **(A)** Left panel: hPDE3A-ir at E12.5, E14.5, E17.5, P2 and P24 in mouse stomach. Middle left panel: KIT-ir at same ages. Middle right panel: αSMA-ir. Right panel: merged images with PDE3A-ir in green, KIT-ir in magenta, αSMA-ir in red and DAPI nuclei counterstain in blue. Confocal microscopy. Scale bar = 50 μm. **(B)** Summary of PDE3A, KIT and αSMA expression in the KIT+ mesenchymal layer during mouse stomach development. hPDE3A-ir was present from E14.5, when differentiation of the mesenchymal precursors started. hPDE3A-ir was still present at P2 in the differentiated SMC and ICC. By P24, PDE3A-ir was only present in the KIT+ ICC.

Having confirmed the presence of PDE3A in adult mouse ICC, PDE3A expression was analyzed during mouse gut development, with emphasis on the mesenchymal ICC progenitors. At embryonic day (E) 12.5, no PDE3A-ir was detected in the layer of KIT+ mesenchymal precursor cells. The smooth muscle cell (SMC) marker αSMA-ir was detected in the developing circular smooth muscle layer but not in the outer layer of KIT+ progenitors. By E14.5, PDE3A-ir was detected in all KIT+ cells and in the newly differentiating KIT+/ αSMA+ cells. At E17.5, as differentiation of the ICC network and the longitudinal smooth muscle layer continued, KIT-ir ICC lost αSMA-ir and the future SMC lost KIT-ir, while PDE3A-ir remained present in both cell types. After birth, PDE3A-ir was still detected in KIT-ir ICC and in the longitudinal SMC at postnatal day (P) 2, to finally become restricted to ICC only by P24 (Figure 2A). No PDE3A-ir was detected in the circular smooth muscle cells at any time point of development. Figure 2B summarizes our data: PDE3A expression appeared around E14.5, when the KIT-ir mesenchymal precursors started to differentiate into ICC and SMC. PDE3A expression persisted in both cell types shortly after birth but restricted to the KIT+ ICC by P24 and at adulthood.

### ICC density is reduced in PDE3A^-/-^ mice

**Figure 3:**
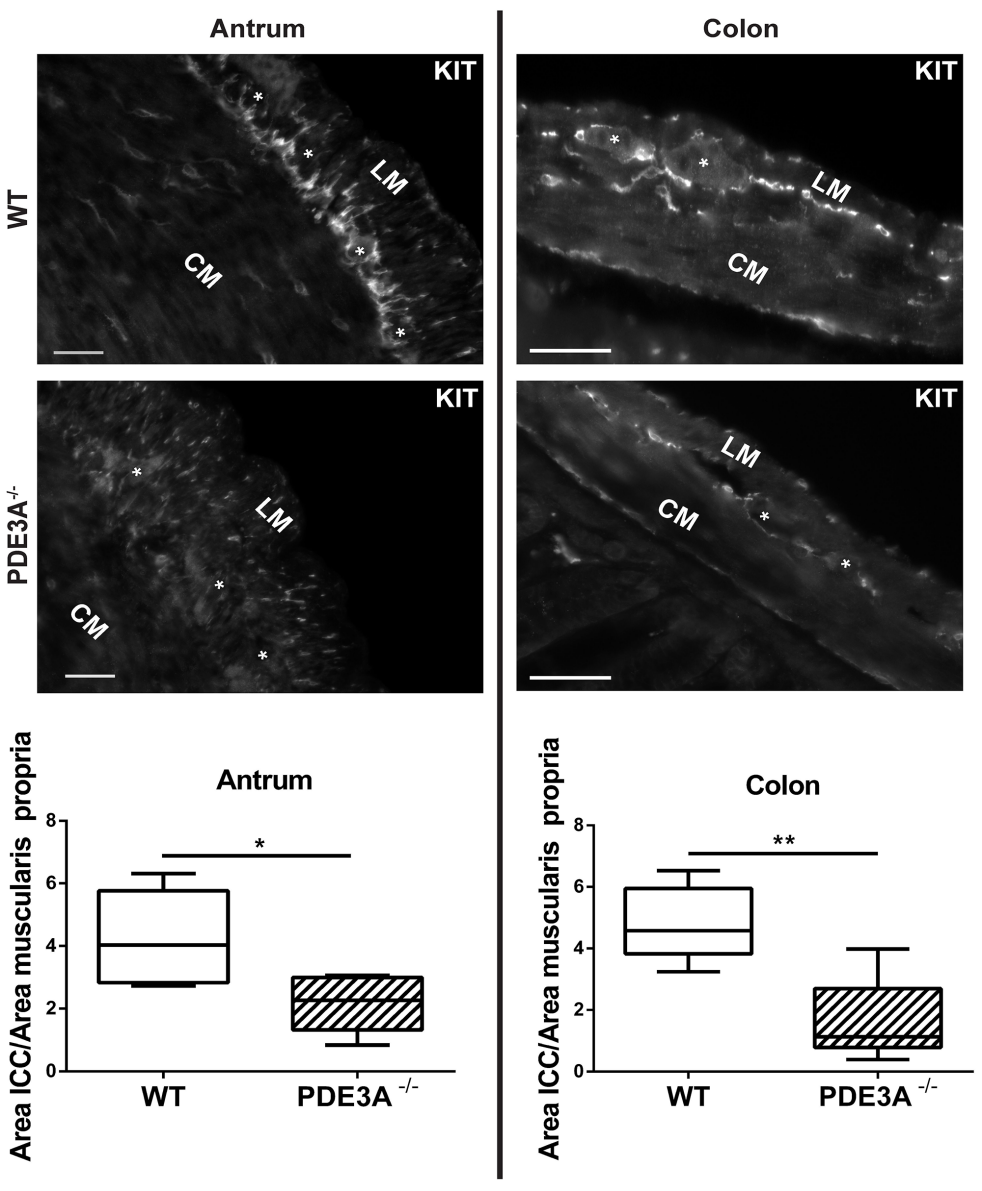
ICC network density is halved in adult PDE3A^-/-^ mouse antrum and colon Left panel, top: representative images of KIT-ir in adult WT and PDE3A^-/-^ mouse antrum. Bottom: ratio of KIT-ir ICC in the muscularis propria of mouse WT and PDE3A^-/-^. Right panel, top: representative images KIT-ir in adult WT and PDE3A^-/-^ mouse colon. Bottom: ratio of KIT-ir ICC in the muscularis propria. Abbreviations: LM: longitudinal muscle layer, CM: circular muscle layer, *: location of myenteric plexus. Widefield microscopy. Scale bar = 50μm. Data presented as mean +/- SEM. P-values (Mann-Whitney t-test) *: p {less than or equal to} 0.05, **: p {less than or equal to} 0.01.

As PDE3A expression occurs throughout the embryonic differentiation of gut mesenchymal precursor cells, we hypothesized that PDE3A might be important for ICC development. We quantified the ICC network density in adult WT and PDE3A^-/-^ mice in antrum and colon, using KIT-ir as fiducial ICC marker. In both antrum and colon, KIT-ir cells were present in all expected locations of the muscularis propria but their density was significantly decreased in PDE3A^-/-^ mice compared to WT littermates (Figures 3A and 3B). The histograms of pixel intensities for KIT staining were similar in WT and PDE3A^-/-^, indicating that the reduction in KIT+ ICC density observed in PDE3A^-/-^ gut cannot be attributed to lower KIT intensities in that genotype (Supplementary Figure 3). No obvious alteration in the enteric nervous system was observed in PDE3A^-/-^ gut (data not shown).

We concluded that PDE3A plays a role in the development or homeostasis of ICC as demonstrated *in vivo*.

### PDE3A is expressed in human ICC and in human gastrointestinal tumors (GIST)

**Figure 4:**
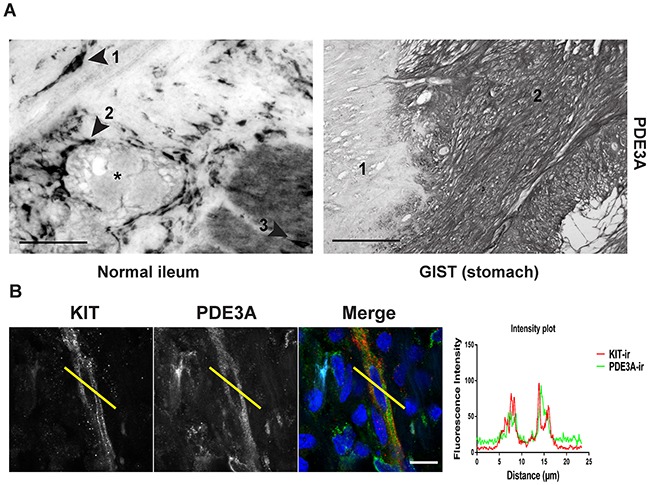
PDE3A is expressed in human ICC and GIST **(A)** Immunohistochemistry. Upper panel: hPDE3A-ir in transversal section of normal human ileum. Arrowhead 1: spindle shaped cell in the circular muscle layer. Arrowhead 2: Cells around the myenteric plexus (highlighted with a *). Arrowhead 3: cell within the longitudinal muscle layer. Lower panel: hPDE3A-ir in GIST (2) adjacent to normal human stomach tissue (1). Widefield microscopy. Scale bars = 50μm and 100μm, respectively. **(B)** Immunofluorescence. KIT-ir in red and hPDE3A-ir in green in human ileum ICC. Intensity plot showing the localisation of hPDE3A-ir in a KIT-ir ICC. Confocal microscopy. Scale bar = 10μm.

As PDE3A appeared to play a role in mouse ICC development or maintenance (Figures 3A and 3B), we asked whether PDE3A was expressed in human ICC and in ICC-derived GIST. In normal human ileum, PDE3A-ir was detected in spindle-shaped cells within the circular muscle layer, around myenteric plexus and within the longitudinal muscle layer, corresponding to the expected locations for ICC (Figure 4A). A double IF confirmed that PDE3A-ir was indeed present in the KIT-ir ICC in normal human ileum (Figure 4B).

**Figure 5:**
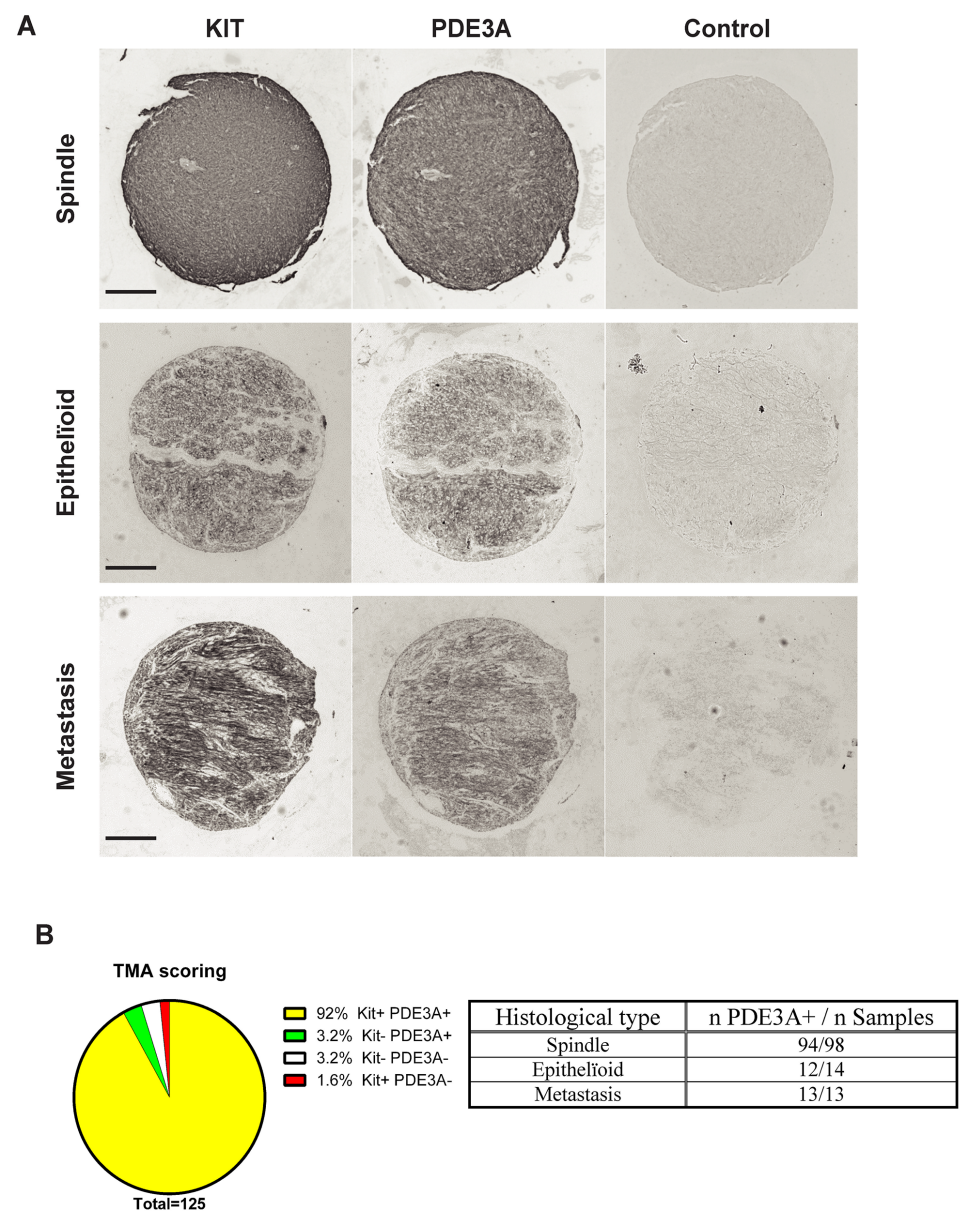
PDE3A-ir in most human GIST irrespective of the histological subtype **(A)** Immunohistochemistry. Examples of hPDE3A-ir in spindle shape, epithelïoid and metastatic human GIST. Widefield microscopy. Scale bar = 200μm. **(B)** Strong correlation (P-value = 0.0001 (Fisher's exact test)) between PDE3A-ir and KIT-ir in a pool of human GIST TMA. (See Supplementary Tables 4 and 5 for details).

In a pilot study, strong PDE3A-ir was detected in all four different GIST cases (Supplementary Table 6). This prompted us to assess PDE3A-ir in a broader range of GIST material. PDE3A and KIT immunohistochemistry was performed on two independent cohorts of GIST tissue microarrays (TMA) – Supplementary Tables 4 and 5. On a total of 125 GIST samples analyzed, PDE3A-ir was detected in 119 out of 125 (92 %) KIT+ GIST samples, indicating a strong correlation between PDE3A and KIT expression in those tumors. PDE3A-ir was found in both spindle and epithelïoid GIST types and in all 5 GIST metastasis as well (Figures 5A and 5B). We concluded that PDE3A-ir is a *bona fide* marker for the KIT-ir GIST and that it might be important for GIST physiology. It therefore represents a potential new therapeutic target in GIST.

### PDE3A is expressed in the GIST882 human cell line

To explore *in vitro* the molecular mechanisms involving PDE3A in human GIST, we used the STI-571 sensitive GIST882 human cell line [22], which harbors an homozygous K-to-E mutation at position 642 in *KIT* exon 13, similar to the mouse Kit^K641E^ mutation [12].

**Figure 6:**
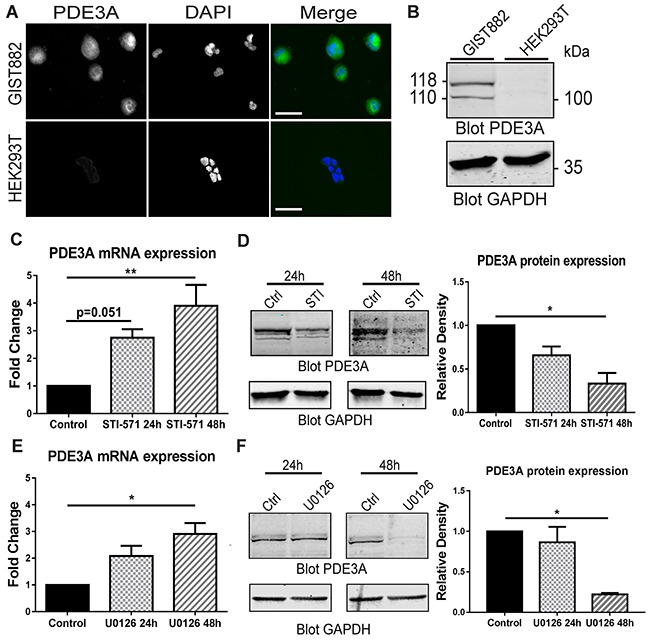
PDE3A expression in the human GIST882 cell line is modulated by KIT and MEK/ERK inhibition **(A)** Immunofluorescence. hPDE3A-ir and DAPI nuclear counterstain in GIST882 and HEK293T cells. hPDE3A-ir was detected in GIST882 while no signal was detected in HEK293T. Widefield microscopy. Scale bar = 50μm. **(B)** Western blot of GIST882 and HEK293T cells probed with anti-hPDE3A and anti-GAPDH as loading control. Bands at 118 kDa and 110 kDa were observed in GIST882 lanes while no band was present in HEK293T lanes. 50μg protein/lane. **(C)** qPCR of GIST882 cells treated with 1μM of the KIT inhibitor STI-571 for 24H and 48h. PDE3A mRNA expression increased significantly after 48h KIT inhibition. **(D)** Left panel: Western blot of GIST882 cells treated for 24h and 48h with 1μM STI-571 probed with anti-hPDE3A and anti-GAPDH antibodies. Right panel: Quantification of PDE3A normalized to loading control GAPDH. PDE3A protein expression was significantly reduced after 48h KIT inhibition. 50μg protein/lane. **(E)** qPCR of GIST882 cells treated with 10 μM of the MEK/ERK inhibitor U0126 for 24h and 48h. PDE3A mRNA expression increased significantly after 48h MEK inhibition. **(F)** Left panel: Western blot of GIST882 cells treated for 24h and 48h with 10μM U0126 probed with anti-hPDE3A and anti-GAPDH antibodies. Right panel: Quantification of PDE3A normalized to loading control GAPDH. PDE3A protein expression was significantly reduced after 48h MEK/ERK inhibition. 100μg protein/lane. Data presented as mean+/- SEM. P-values (Kruskal-Wallis followed by Dunn's test). *: p {less than or equal to} 0.05, **: p {less than or equal to} 0.01.

We first confirmed the presence of PDE3A-ir in GIST882 cells by immunofluorescence. HEK293T were used as negative control for PDE3A expression (Figure 6A). A Western blot was performed on GIST882 and HEK293T extracts (Figure 6B). 115 kDa and 118 kDa bands were immunodetected in GIST882 extracts probed with hPDE3A antibody, while no band was detected in HEK293T extracts.

### KIT receptor activity modulates PDE3A expression through MAPK/ERK pathway at transcriptional and protein level in GIST882 cells

As constitutive tyrosine kinase activity of the mutated KIT RTK is required for GIST822 survival and proliferation [22], we asked whether KIT activity and its downstream signaling pathways affected PDE3A expression. qPCR of GIST882 cells treated with 1μM of the KIT tyrosine kinase inhibitor STI-571 showed a time-dependent significant increase in PDE3A mRNA level after 48H of treatment (Figure 6C). Conversely, at the protein level, an opposite effect was observed by Western blotting, with a significant decrease of PDE3A in GIST882 cells treated for 48H with 1μM STI571 (Figure 6D). Similarly, treatment with 10 μM of the MEK inhibitor U0126 showed a time-dependent increase of PDE3A mRNA level after 48h (Figure 6E) while PDE3A protein level decreased after 48h treatment (Figure 6F). No changes of PDE3A mRNA and protein levels were observed after 24h and 48h hours treatment with 7.5μM of the AKT inhibitor, (not shown).

We concluded that the MAPK/ERK pathway downstream of KIT appears to control in opposite directions PDE3A transcript and protein levels. (Supplementary Figure 2).

### The PDE3 inhibitor cilostazol decreased GIST882 viability and synergized with STI-571

**Figure 7:**
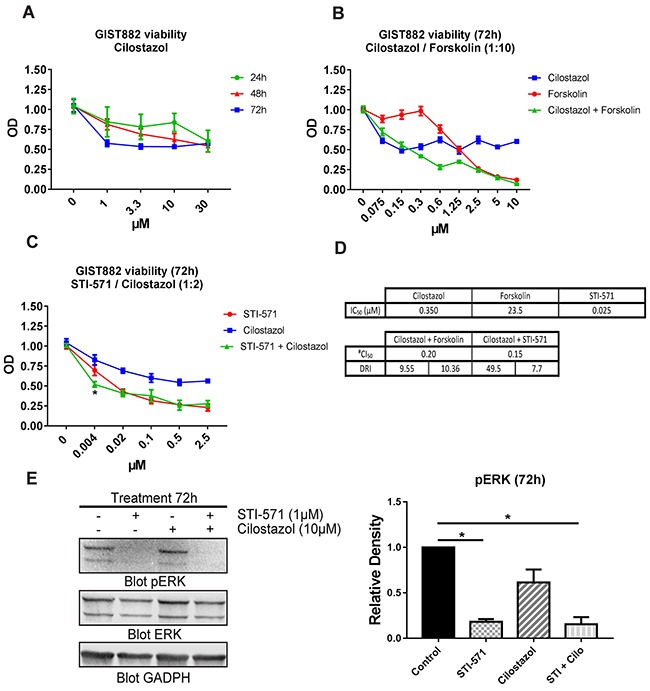
The PDE3 inhibitor cilostazol reduced GIST882 viability and synergized with STI-571 **(A)** WST-1 viability assay. GIST882 were treated with a concentration range of cilostazol for 24h, 48h and 72h prior to addition of WST-1 reagent. Cell viability reduction was time and concentration dependent. **(B)** GIST882 were treated with forskolin (0 to 100μM), cilostazol (0 to 10μM) and combination of the two at a 1:10 ratio (e.g: 1.25μM cilostazol+ 12.5μM forskolin) for 72h. **(C)** GIST882 were treated with STI-571 (0 to 2.5μM), cilostazol (0 to 5μM) and combination of the two at a 1:2 ratio (e.g: 0.5 μM STI-571 + cilostazol 1μM) for 72h. **(D)** Table shows calculated IC50 for cilostazol, forskolin and STI-571 and their respective dose reduction index (DRI). The CI50 value of 0.20 and 0.15 indicates synergism of cilostazol with forskolin and with STI-571, respectively, on cell viability reduction. #(Chou-Talalay's Combination Index for 50% of the effect). Mean values from five independent experiments. Data presented as mean +/- SEM. P-value (2 way ANOVA and Tukey's post- test) *: p {less than or equal to} 0.05. **(E)** Left panel: Western blot of GIST882 cells treated for 72h with STI-571 (1μM), cilostazol (10μM) and STI-571+cilostazol (1μM:10μM) probed with anti-pERK, anti-total ERK and anti-GAPDH. 100μg protein/lane. Right panel: pERK levels were significantly reduced in STI-571 and STI-571+cilostazol conditions. Data presented as mean +/- SEM. P-values (Kruskal-Wallis followed by Dunn's test). *: p {less than or equal to} 0.05.

As KIT activity modulates PDE3A expression in GIST882, we also assessed the effect of the PDE3 specific inhibitor cilostazol [23] on GIST882 cell viability. GIST882 cells treated for 24h, 48h and 72h with cilostazol showed a time and concentration dependent decreased cell viability. After 72h, cell viability was reduced of approximately 50% at all tested concentrations (Figure 7A).

As PDE3 inhibition reduced GIST882 viability, we have tested the combined inhibition of KIT and PDE3. Firstly, we determined the dose response curve and IC_50_ value of each drug for 72h. Then we combined STI-571 and cilostazol at a concentration ratio of 1:2 for 72h (Figure 7C). We applied the Chou-Talalay's method to calculate the Combination Index which indicates if two drugs have an antagonist (>1), additive (=1) or synergistic effect (<1). The CI_50_ of 0.15 (Chou-Talalay's Combination Index for 50% of the effect) indicated a synergy between cilostazol and STI-571 on cell viability reduction. The dose reduction index (DRI), meaning how much the dose of each drug can be reduced at a given level (here, 50% of the effect), was 7.7 for STI-571 and 49.5 for cilostazol. (Figure 7D). Next, we have tested whether increased cAMP level by the adenylyl cyclase activator forskolin would also affect GIST882 viability. Forskolin reduced GIST882 cells viability (IC_50_: 23.5μM) and synergized with cilostazol at a concentration ratio of 1:10 (Figure 7B): Chou-Talalay's CI_50_ 0.2, DRI 10.36 for forskolin and 9.55 for cilostazol (Figure 7D).

Finally, we checked whether inhibition of PDE3A affected the phosphorylation of ERK (pERK), a marker of activation of the MAP kinase (MAPK) pathway in GIST882 cells. pERK was decreased in GIST822 treated for 24h and 48h with STI-571 1μM, and combination of STI-571 1μM + cilostazol 10μM, while no significant change was observed for 10 μM cilostazol alone.

### DNMDP, a non-catalytic inhibitor of PDE3A reduced GIST882 viability

A recent predictive chemogenomics screen in a large number of human cell lines by de Waal *et al*., identified a novel, non-catalytic, function for PDE3A. The DNMDP compound was found to elicit cytotoxicity selectively for cells expressing both PDE3A and SLFN12, by inducing a neomorphic interaction between the two proteins, as demonstrated in HeLa cells. [18]

**Figure 8:**
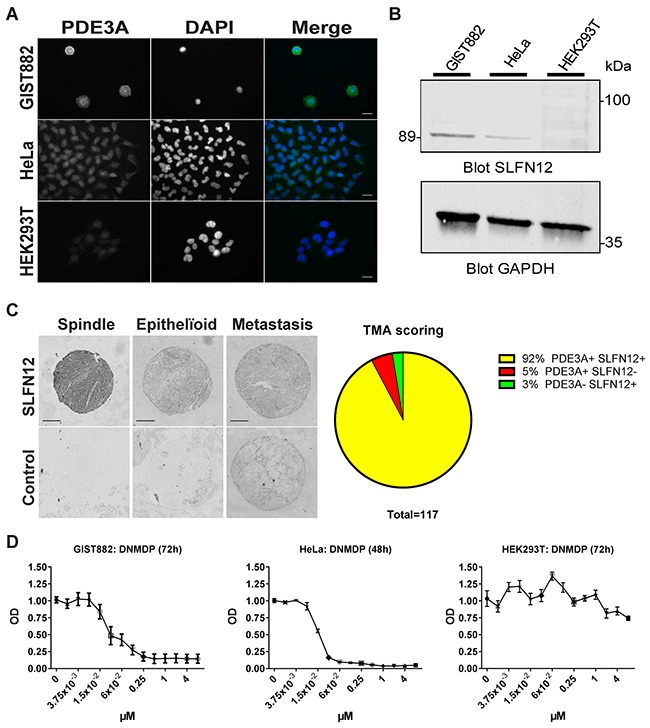
SLFN12 expression in GIST **(A)** Immunofluorescence. SLFN12-ir in GIST882, HeLa and HEK293T cells with DAPI nuclear counterstain. SLFN12-ir was detected in GIST882 and HeLa while no signal was detected in HEK293T. Widefield microscopy. Scale bar = 20μm. **(B)** Western blot of GIST882, HeLa and HEK293T cells probed with anti-SLFN12 and anti-GAPDH as loading control. A 89 kDa band was observed in GIST882 and HeLa lanes while no band was present in HEK293T lane. 100μg protein/lane. **(C)** Right panel: Immunohistochemistry. Representative examples of SLFN12-ir in spindle shape, epithelïoid and metastatic human GIST. Widefield microscopy. Scale bar = 200μm. Left panel: TMA scoring. **(D)** WST-1 viability assay. HeLa, GIST882 and HEK293T were treated with a range of DNMDP concentration. DNMDP markedly reduced viability of GIST882 (IC50: 27 nM) and HeLa cells (IC50: 22nM) but not of HEK293T. Mean values from three independent experiments.

Therefore, we assessed the expression of SLFN12 in GIST882 cells. SLFN12-ir was detected in HeLa, used as positive control, and in GIST882 cells, while no SLFN12-ir was detected in a negative control cell line, HEK293T (Figure 8A). Western blot for SLFN12 expression, showed a ~89 kDa band in GIST882 and HeLa extracts, but no band in HEK293T extract (Figure 8B).

As SLFN12 was expressed in the GIST882 cell line, we further assessed the expression of SLFN12 in human GIST TMA. Immunohistochemistry showed SLFN12-ir in 92% (108 out of 117) of GIST samples, which were also PDE3A-ir, regardless of their histological subtypes (Figure 8C). Having established the expression of PDE3A and SLFN12 in GIST882 cells, the effect of DNMDP was tested on cell viability. GIST882 showed a concentration-dependent reduction of viability 72h after treatment with DNMDP (IC_50_: 27 nM), similar to HeLa cells treated for 48h (IC_50_: 22 nM), while HEK293T cells deficient for PDE3A and SLFN12 did not respond to DNMDP (Figure 8D).

## DISCUSSION

We previously identified PDE3A as a novel ICC marker [16]. In this study, evidence is presented to establish that PDE3A is not merely a marker but also an actor in ICC and GIST biology. We first addressed whether PDE3A would play a role in ICC development in mice *in vivo* and subsequently studied the role of PDE3A in human GIST cell models.

Chen *et al*. [24] used FACS to isolate ICC from neonatal mouse intestine to study their expression profile by microarray analysis. In contrast to other PDEs – including PDE3B -, PDE3A was highly expressed in the two classes of ICC present in the intestine, ICC-MP and ICC-DMP, with a 6- and 4.3-fold higher expression as compared to surrounding tissue, respectively. We reported that PDE3A was amongst the genes upregulated in the mouse W^K641E^ GIST model characterized by a marked hyperplasia of ICC [11]. PDE3A-ir was shown to be a specific immunomarker for ICC in mouse and human gut [16]. These findings raised the question whether PDE3A could be not only a marker, but also an actor in ICC development.

Here, we provided original data over PDE3A expression during the differentiation process of the mouse gut mesenchyme into ICC or SMC [25]. KIT+ mesenchymal progenitor started to express PDE3A from the beginning of their differentiation, around E14.5, and persisted in both ICC and SMC throughout embryonic life. Shortly after birth, PDE3A-ir vanished in SMC, while it persisted in KIT+ ICC throughout life. We also found that adult PDE3A^-/-^ mice (lacking PDE3A expression) exhibit a significantly reduced density of KIT+ ICC along the gut.

Taken together, our observations suggest that PDE3A, although not essential for ICC occurrence, plays nevertheless a significant role in the development of the KIT+ ICC lineage. The ICC reduction observed in PDE3A^-/-^ mice also indicates that PDE3B cannot compensate for the absence of PDE3A in ICC development. We next addressed the role of PDE3A in human GIST

KIT is widely regarded as the fiducial marker for ICC and GIST in human [26] while additional markers such as anoctamin channel 1 (ANO1, a.k.a. DOG-1, TMEM16A), might prove very useful diagnostic tools in KIT negative GIST [27], [28]. Here we confirmed that in the normal human gut PDE3A-ir was present in the KIT-ir ICC, and in no other cell type in the muscularis propria. PDE3A-ir was found in most GIST samples examined, regardless of their histological type. PDE3A-ir thus appears as a prospective novel marker for GIST diagnosis. Further assessment should be carried out on larger cohorts, including a larger number of samples from KIT negative GIST and other mesenchymal gut tumors, to precisely determinate the place of PDE3A-ir in clinical pathology.

In the STI-571 sensitive human cancer cell line GIST882, PDE3A expression was detected by IF and two PDE3A protein isoforms identified by Western blotting at an apparent molecular weight of 115 and 118 kDa, respectively. Several protein isoforms at apparent molecular weight between 100 and 130 kDa for PDE3A have been reported in various human cell types [29] [30] [18]. Although the different isoforms may play different functions depending of the cellular context, in human myocardium, the 136 kDa, 118 kDa and 94 kDa isoforms have similar K(m) and k(cat) values for cAMP, are equal in their sensitivities to inhibition by cGMP and cilostazol [31].

PDE3A RNA expression in GIST882 cells was confirmed by qPCR, highlighting the dynamic interplay between KIT receptor activity and PDE3A expression: inhibition of KIT activation led to a decrease in PDE3A protein level but also to an upregulation of PDE3A gene expression. The MAPK/ERK pathway, a major player in KIT signaling [1], [32], appears to be the main pathway involved, as the MEK inhibitor U0126 replicated the effect of STI-571 on PDE3A protein level and gene expression. Conversely, inhibition of the AKT pathway had no effect. KIT inhibition beyond 48 hours lead to massive cell death and precluded observation at later time points. The mechanisms underlying PDE3A protein turn-over, and controlling PDE3A gene expression (regulation of PDE3A promoter activity or stability of PDE3A transcripts) remain to be unraveled.

PDEs are already established therapeutic targets in various human diseases, such as cardiac failure, pulmonary hypertension and erectile dysfunction [reviewed in 15]. In various cancer cell lines *in vitro*, inhibition of the enzymatic activity of PDE1 or PDE4 isoenzymes, increases cAMP level, leading to growth arrest or apoptosis [33],[34],[35] while the selective PDE3 inhibitor cilostazol has been reported, in a single report, to reduce colon cancer cell motility [36].

Our present data shed new light on the role of PDE3A in GIST. The PDE3 inhibitor cilostazol [37] significantly reduced GIST882 cells viability (cilostazol IC_50_ 0.35 μM), albeit to a lesser extent than STI-571 – 50% vs 75 % cell viability reduction at 72h, respectively (Figure 7C).

Cilostazol is a selective inhibitor of the catalytic activity of type 3 PDEs, PDE3A and PDE3B [37]. Both appear to be expressed in ICC [16] and in GIST. As discussed above, PDE3A – and not PDE3B – was prominent in mouse ICC and data mining using MERAV (Metabolic gEne RApid Visualizer) http://merav.wi.mit.edu/, that allows comparison of gene expression across human tissue and cell types [38], indicates that PDE3A appeared to be more highly expressed than PDE3B in primary GIST tumors (Supplementary Figure 4). Although a role for PDE3B cannot be strictly excluded, it appears that the reduction in GIST882 cell viability observed with cilostazol likely results from an inhibitory effect of PDE3A rather than PDE3B.

Interestingly, cilostazol synergized with the KIT inhibitor STI-571 (Chou-Talalay's CI_50_ <1) to reduce GIST882 cells viability. That synergism opens the perspective of clinical benefit. Both drugs are already in clinical use and clinical studies could readily test the hypothesis that the combination of the two may reduce side effects of high dose STI-571 - or enhance the efficacy of a given dose of STI-571.

Forskolin, an ubiquitous activator of eukaryotic adenylyl cyclase, acts by raising intracellular cAMP level [39]. Forskolin reduced GIST882 cells viability and synergized with cilostazol (Figure 7B) as indicated by the Chou-Talalay's CI_50_ <1 (0.2). Our data thus suggest that cilostazol, in line with its well-established role of selective PDE3 catalytic inhibitor, reduced GIST882 cell viability by inhibition of the catalytic function of PDE3A.

Besides the selective inhibition of PDE3 catalytic activity, various non-catalytic effects of cilostazol have been reported before, e.g. the activation of AMP-activated protein kinase in vascular smooth muscle cells [40], the anti-inflammatory effect in microglial cells [41], inhibition of low-density lipoprotein uptake in mouse peritoneal macrophages [42] or inhibition of interleukin-1-induced ADAM17 expression in vascular smooth muscle cells [43]. In several studies, the MAPK/ERK pathway appeared to be involved in the non-catalytic effect of cilostazol [41], [43]. However, our results showed that 10 μM cilostazol did not alter significantly ERK phosphorylation (Figure 7E), indicating that, in contrast with the KIT inhibitor STI-571, the reduction of GIST882 cells viability by cilostazol alone does not appear to be mediated by a reduction of activation of the MAPK/ERK pathway.

Several interactors of PDE3A have indeed been identified in other cell types [17], e.g. 14-3-3 proteins [20], and PP2A [21]. One possibility to consider is that cilostazol may stabilize a conformation of PDE3A that triggers protein:protein interaction that accelerates its degradation.

The recent predictive chemogenomics study by de Waal *et al*. identified that the DNMDP compound exerted a cytotoxic action only on cells expressing both PDE3A and SLFN12. Data mining also indicated that co-occurrence of PDE3A and SLFN12 is rare in normal tissues, while their co-occurrence was found in a subset of cancer cell lines, opening the perspective of a novel class of cancer-selective cytotoxic compounds. The molecular mechanism by which the PDE3A-SLFN12 interaction induced by DNMDP leads to cytotoxicity remains to be unveiled [18]. Here we confirmed de Waal *et al*. observation of DNMDP cytotoxicity in HeLa cells and found that GIST882 cells, which, like HeLa cells, express both PDE3A and SLFN12 are similarly affected by DNMDP cytotoxicity. Furthermore, we found that SLFN12-ir was present in most (108 out of 117, 92%), PDE3A-ir GIST samples.

In summary, we have unraveled the importance of PDE3A in mouse ICC development and shed new light on PDE3A and the PDE3A-interacting compounds cilostazol and DNMDP in human GIST. The reduction of cell viability by cilostazol, its synergism with KIT inhibition by STI-571 and the DNMDP cytotoxicity observed in GIST882 cells *in vitro* on one hand, and the broad expression of PDE3A and SLFN12 in human GIST tissue arrays on the other, make of PDE3A a novel appealing prospect in the ongoing quest for GIST targeted therapy.

## MATERIALS AND METHODS

### Ethics statement

Study of Kit^K641E^ and WT mice was approved (protocol LA1230331-491N) by the Ethics Committee for Animal Well-Being of the Faculty of Medicine, Université Libre de Bruxelles (ULB).

The collection and analysis of human tissue samples has been conducted in accordance with the ethical standards and according to the Declaration of Helsinki and according to national and international guidelines and has been approved (protocol P2010/106) by the Institutional Medical Ethics Committee of Erasmus Hospital and Faculty of Medicine, Université Libre de Bruxelles, Brussels, Belgium.

### Animals

Five months old pregnant 129/Sv wild type mice were sacrificed by cervical dislocation and decapitation. Embryos (E12.5, E14.5 and E17.5) were quickly removed from uterus, placed in cold PBS 10mM pH7.4 and decapitated. Young wild type mice (P2 and P24) and adult WT and PDE3A^-/-^ mice [19] were sacrificed by cervical dislocation and decapitation. Small intestine, colon and stomach were promptly removed and the gastric antrum was delineated from corpus based on visual landmarks on the serosa. Luminal content was gently emptied and surrounding tissues (e.g. mesenteric fat) were carefully removed by sharp dissection without damaging the serosa.

### Cell line and drugs

The human GIST cell line GIST882 was kindly provided by Dr. Jonathan A. Fletcher, Harvard Medical School, Boston, MA, USA. Cells were cultured at 37°C in DMEM (GIBCO, CA, USA) supplemented with 10% FBS, 2% penicillin/streptomycin. HeLa cells were cultured at 37°C in DMEM (GIBCO, CA, USA) supplemented with 10% FBS, 2% penicillin/streptomycin. HEK293T were cultured at 37°C in DMEM + GlutaMAX–I (GIBCO, CA, USA) supplemented with 10% FBS, 2% penicillin/streptomycin. Cilostazol, U0126 and forskolin were purchased from Sigma, St. Louis, MO, USA, STI-571 was purchased from LC Laboratories, Woburn, MA, USA, DNMDP was purchased from Aobious Inc, Gloucester, MA, USA and Akti was purchased from Merck, Darmstadt, Germany.

### Human tissue and human GIST tissue microarrays (TMA)

Two independent cohorts of GIST microarray slides of formalin-fixed, paraffin-embedded (FFPE) material were used.

The SuperBiochips GIST TMA (#DAA1) was purchased from Super BioChips laboratories, Seoul, South Korea. It contained 50 FFPE human GIST tissue specimens and 9 matched normal gut tissue specimens.

The second TMA, purchased from CMMI-DiaPath (Center for Microscopy and Molecular Imaging, Gosselies, Belgium), originates from the Department of Pathology, Erasmus Academic Hospital, Université Libre de Bruxelles, Brussels, Belgium. It contained a total of 75 FFPE human GIST tissue specimens including 5 metastatic specimens. Clinicopathological features are given in Supplementary Tables 4 and 5, respectively.

For immunohistochemistry, FFPE slides were rehydrated through phenol and graded alcohol solutions then heated at 90°C in 0.1M EDTA, 0.05% Tween 20 (pH 8.0) antigen retrieval solution for 20 min to achieve PDE3A epitope unmasking and in 0.01M citrate buffer (pH 6.0) for SLFN12 epitope unmasking. Slides were then cooled at room temperature (RT) for 10 min before being put in a 0.1% H_2_O_2_/methanol solution for 30 min to block endogenous peroxidase. After washing, primary antibodies diluted in a TBS-Triton X-100 0.1% and 1% NHS solution were incubated overnight at RT in a humid chamber. Sections were rinsed and incubated with a secondary biotinylated antibody for 1h then with an ABC solution (ABC kit standard PK-4000; Vector Laboratories, Burlingame, CA, USA) for 1h. Revelation with nickel-enhanced DAB (DAB-Ni) was performed at RT for 5–10 min, resulting in a black precipitate. The DAB-Ni solution was prepared by dissolving 0.06 g of nickel ammonium sulphate (Fluka, Buchs, Switzerland) and 2 mg of DAB (Sigma-Aldrich) in 10 ml of 0.05 M Tris/HCl, pH 8. Immediately before use, 1 μl of 30% H2O2 (Merck, Darmstadt, Germany) was added. Spots were evaluated by three examiners blinded for clinicopathological information. Immunoreactivity was considered as positive when signal was above signal in the negative control. Antibodies used are listed in Supplementary Table 1.

### Immunofluorescence (IF)

Mouse tissues and whole embryos were fixed for 24h at 4°C in freshly prepared 4% paraformaldehyde, PH 7.4, and cryopreserved in sucrose solutions (10%, 20%, 30% w/v in water), overnight (o/n) each, embedded in OCT (Sakura Finetec Europe, Leiden, the Netherlands) and frozen at -80°C. Sections (16μm thick) were cut on a CM3050S cryostat (Leica Microsystems GmbH, Wetzlar, Germany), collected on Superfrost Plus glass slides (Thermo Scientific, Waltham, MA, USA) and stored at -20°C until use.

Cells were washed with 0.01M PBS pH7.4 and fixed for 30 min in fresh 4% paraformaldehyde, pH 7.4 then washed two times with 0.01M TBS, pH7.4 and stored in 0.01M TBS/Sodium azide 0.1% solution until use.

For immunostaining, slides were brought to RT, permeabilized and blocked for 1hin 0.01M TBS pH 8.2 containing 0.1% Triton X-100 (Sigma, Saint Louis, MO, USA) and 10% normal horse serum (NHS). Primary antibodies were diluted in a TBS-Triton X-100 0.1% and 1% NHS solution and incubated overnight at RT in a humid chamber. Slides were washed in TBS and incubated at RT for 1 hour in TBS containing the secondary antibodies. Slides were washed and mounted using Glycergel (Dako, Glostrup, Denmark) + 2.5% DABCO (Sigma). Antibodies used are listed in Supplementary Table 1.

Slides were observed and imaged on an AxioImager Z1 fluorescent microscope (Zeiss, Jena, Germany), using a Plan Apochromat 20x/0.8 or EC Plan NeoFluar 40x/0.75 objective. Excitation was provided by a HBO 105W lamp. Band pass filters sets #49, #38; and #43 (Zeiss) were used to detect blue, green and red fluorochromes, respectively. Images (1388 by 1040 pixels, pixel size (x-y): 0.32 micron by 0.32 micron) were acquired sequentially with an AxioCamMRm camera (Zeiss) as 3 × 12 bit RBG proprietary zvi files. Files were processed with AxioVision 4.6 software (Zeiss). Images were displayed in linear mode with manual contrast adjustment and exported as uncompressed. TIF files. Figures were prepared with Adobe Illustrator.

Quantitation of KIT-ir ICC density was performed using the Fiji software [44] as described previously [16]. Briefly, the plugin “Stich grid of images” [45] was used to assemble images covering the entire circumference of the sample. Next, the boundaries of the αSMA-ir muscularis propria were delineated to extract the region of interest (ROI). The area occupied by the ICC was determined by thresholding the KIT-ir signal within the ROI of the muscularis propria.

### Confocal microscopy

High resolution imaging was performed using a Zeiss LSM780 system fitted on an Observer Z1 inverted microscope equipped with a LD LCI C-Apochromat 40x/1.1W objective (Zeiss). The 488 nm excitation wavelength of the Argon/2 laser, a main dichroic HFT 488 and a band-pass emission filter (BP490-535 nm) were used for selective detection of the green fluorochrome. The 543 nm and 633 nm excitation wavelength of the HeNe1 laser, a main dichroic HFT 488/543/633 and a long-pass emission filter (BP553-624 nm) (BP652-735 nm) were used for selective detection of the red fluorochrome and far-red fluorochrome. A 405 nm blue diode, a main dichroic HFT 405 and a band-pass emission filter (BP415-468 nm) were used for selective detection of the DNA counterstain. Single optical sections were acquired sequentially with a zoom factor of 1 and optimal (1 Airy unit) pinhole (scaling (x-y-z): 0.21 × 0.21 × 0.53 micron) and stored as 8-bit proprietary czi files. Single plane images were displayed using Zen2010 software (Zeiss) and exported as 8 bits uncompressed TIF images.

### Western blot

Cell samples were lysed for 2h at 4°C in lysis buffer containing 0.01M Tris-HCl (pH 7.4), 0.15M KCl, 0.1M NaF, 0.002M EDTA, 0.012M β-mercaptoethanol, 0.5% Nonidet P-40 and a cocktail of protease inhibitors (leupeptin 0.01mg/mL; 0.001M Na_3_VO_4_; Pefabloc 0.3mg/mL; 0.01μM okadaic acid). Protein were solubilized in sample buffer, heated at 95°C for 1 min, separated by SDS-PAGE on 10% polyacrylamide gel and transferred on a 0.2 μm nitrocellulose membrane. Primary antibodies raised in different species and secondary antibodies coupled with different fluorochromes, were sequentially combined to specifically label one marker in green (800 Li-Cor), the other in red (680 Li-Cor). The Odyssey™ imaging system (LI-COR Biotechnology, Lincoln, NE, USA) and the Azure c500 imaging system (Azure Biosystems, Dublin, CA, USA) was used to quantify the signals. Antibodies used are listed in Supplementary Table 2.

### Real time quantitative PCR (qPCR)

A minimum of five different RNA samples from GIST882 non-treated, treated 24h with 1 μM STI-571 and 48h 1μM STI-571 were used. Total RNA was extracted using RNeasy MiniKit (Qiagen, Valencia, CA, USA) according to the manufacturer's instructions. Genomic DNA was removed using the RNase- Free DNase set (Qiagen). RNA was reverse transcribed with 200 units of M-MLV Reverse Transcriptase (Invitrogen, Eugene, Oregon, USA) in a reaction containing 1μg of random primers (Amersham Bioscience, Piscataway, NJ, USA), 0.01M each dNTP, 1x First-Strand buffer and 0.1M dithiothreitol followed by heat deactivation. The cDNA reverse transcription product was amplified with specific primers (Supplementary Table 2) by qPCR using SYBR Green chemistry on a 7500 Real-time PCR system (Applied Biosystems, Foster City, CA, USA). Identical thermal profile conditions, namely 95°C for 10min, then 40 cycles of 95°C for 15sec and 60°C for 1min were used for all primer sets. Emitted fluorescence was measured during annealing/ extension phase and amplification plots were generated using the Sequence Detection System. Transcriptional quantification relative to GAPDH and β-actin reference genes was performed using qBase+ software (Biogazelle, Zwijnaarde, Belgium). Primers used are listed in Supplementary Table 3.

### Cell viability assay

Cell viability was measured using a WST-1 assay (Roche, Indianapolis, IN, USA). HEK293T, HeLa and GIST882 were seeded in 96-well plates (TPP Techno Plastic Products AG, Trasadingen, Switzerland) at a concentration of 10.000 cells/well supplemented with 100μl of medium 48h before drug treatments. After drug treatment, 10μL of WST-1 reagent was added and plates were incubated for 2h at 37°C. Absorbance was measured at 450nm on a plate reader (iMark Microplate Absorbance Reader, BioRad, Hercules, CA, USA).

The CalculSyn v2.0 software (http://www.combosyn.com) was used to calculate the Chou-Talalay's Combination Index for 50% of the effect (CI_50_) [46]. Drug ratio was based on drugs IC_50_ as recommended by Chou [46].

### Statistics

Statistical analysis was performed with Prism 7 software (GraphPad Software, Inc., La Jolla, CA, USA), using Mann–Whitney test to compare mouse genotype, Kruskal-Wallis test with Dunn's post hoc test for western blot experiments, 2-way ANOVA with Tukey's post-test for cell viability experiments and Fisher's exact test for correlation analysis in TMA. A p-value smaller than 0.05 was regarded as statistically significant. All data are presented as mean +/- SEM.

## SUPPLEMENTARY MATERIALS FIGURES AND TABLES



## References

[1] Joensuu H, Hohenberger P, Corless CL (2013). Gastrointestinal stromal tumour. Lancet.

[2] Demetri GD, von Mehren M, Blanke CD, Van den Abbeele AD, Eisenberg B, Roberts PJ, Heinrich MC, Tuveson DA, Singer S, Janicek M, Fletcher JA, Silverman SG, Silberman SL (2002). Efficacy and Safety of Imatinib Mesylate in Advanced Gastrointestinal Stromal Tumors. N Engl J Med.

[3] Demetri GD, van Oosterom AT, Garrett CR, Blackstein ME, Shah MH, Verweij J, McArthur G, Judson IR, Heinrich MC, Morgan JA, Desai J, Fletcher CD, George S (2006). Efficacy and safety of sunitinib in patients with advanced gastrointestinal stromal tumour after failure of imatinib: a randomised controlled trial. Lancet.

[4] Demetri GD, Reichardt P, Kang Y, Blay J, Rutkowski P, Gelderblom H, Hohenberger P, Leahy M (2013). Efficacy and safety of regorafenib for advanced gastrointestinal stromal tumours after failure of imatinib and sunitinib (GRID): an international, multicentre, randomised, placebo-controlled, phase 3 trial. Lancet.

[5] Wardelmann E, Thomas N, Merkelbach-Bruse S, Pauls K, Speidel N, Reinhard B, Bihl H, Leutner CC, Heinicke T, Hohenberger P (2005). Case Report Acquired resistance to imatinib in gastrointestinal stromal tumours caused by multiple KIT mutations. Lancet Oncol.

[6] Rumessen JJ, Vanderwinden JM (2003). Interstitial cells in the musculature of the gastrointestinal tract: Cajal and beyond. Int Rev Cytol.

[7] Sanders KM, Ward SM, Koh SD (2014). Interstitial cells: regulators of smooth muscle function. Physiol Rev.

[8] Klüppel M, Huizinga JD, Malysz J, Bernstein A (1998). Developmental origin and Kit-dependent development of the interstitial cells of cajal in the mammalian small intestine. Dev Dyn.

[9] Torihashi S, Ward SM, Sanders KM (1997). Development of c-Kit-positive cells and the onset of electrical rhythmicity in murine small intestine. Gastroenterology.

[10] Maeda H, Yamagata A, Nishikawa S, Yoshinaga K, Kobayashi S, Nishi K (1992). Requirement of c-kit for development of intestinal pacemaker system. Development.

[11] Gromova P, Ralea S, Lefort A, Libert F, Rubin BP, Erneux C, Vanderwinden JM (2009). Kit K641E oncogene up-regulates Sprouty homolog 4 and trophoblast glycoprotein in interstitial cells of Cajal in a murine model of gastrointestinal stromal tumours. J Cell Mol Med.

[12] Rubin BP, Antonescu CR, Scott-Browne JP, Comstock ML, Gu Y, Tanas MR, Ware CB, Woodell J (2005). A knock-in mouse model of gastrointestinal stromal tumor harboring kit K641E. Cancer Res.

[13] Omori K, Kotera J (2007). Overview of PDEs and Their Regulation. Circ Res.

[14] Azevedo MF, Faucz FR, Bimpaki E, Horvath A, Levy I, De Alexandre RB, Ahmad F, Manganiello V, Stratakis CA (2014). Clinical and molecular genetics of the phosphodiesterases (pdes). Endocr Rev.

[15] Maurice DH, Ke H, Ahmad F, Wang Y, Chung J, Manganiello VC (2014). Advances in targeting cyclic nucleotide phosphodiesterases. Nat Rev Drug Discov.

[16] Thys A, Vandenberghe P, Hague P, Klein OD, Erneux C, Vanderwinden JM (2015). Hyperplasia of interstitial cells of cajal in sprouty homolog 4 deficient mice. PLoS One.

[17] Corradini E, Klaasse G, Leurs U, Heck AJR, Martin NI, Scholten A, Braumann T, Erneux C, Petridis G, Stohrer WD, Jastorff B, Trong HL, Beier N (2015). Charting the interactome of PDE3A in human cells using an IBMX based chemical proteomics approach. Mol BioSyst.

[18] de Waal L, Lewis TA, Rees MG, Tsherniak A, Wu X, Choi PS, Gechijian L, Hartigan C, Faloon PW, Hickey MJ, Tolliday N, Carr SA, Clemons PA (2015). Identification of cancer-cytotoxic modulators of PDE3A by predictive chemogenomics. Nat Chem Biol.

[19] Masciarelli S, Horner K, Liu C, Park SH, Hinckley M, Hockman S, Nedachi T, Jin C, Conti M, Manganiello V (2004). Cyclic nucleotide phosphodiesterase 3A-deficient mice as a model of female infertility. J Clin Invest.

[20] Pozuelo Rubio M, Campbell DG, Morrice NA, Mackintosh C (2005). Phosphodiesterase 3A binds to 14-3-3 proteins in response to PMA-induced phosphorylation of Ser428. Biochem J.

[21] Beca S, Ahmad F, Shen W, Liu J, Makary S, Polidovitch N, Sun J, Hockman S, Chung YW, Movsesian M, Murphy E, Manganiello V, Backx PH (2013). Phosphodiesterase type 3A regulates basal myocardial contractility through interacting with sarcoplasmic reticulum calcium atpase type 2a Signaling complexes in mouse heart. Circ Res.

[22] Tuveson DA, Willis NA, Jacks T, Griffin JD, Singer S, Fletcher CD, Fletcher JA, Demetri GD (2001). STI571 inactivation of the gastrointestinal stromal tumor c-KIT oncoprotein: biological and clinical implications. Oncogene.

[23] Shintani S, Watanabe K, Kawamura K, Mori T, Tani T, Toba Y, Sasabe H, Nakagiri N, Hongoh O, Fujita S (1985). General pharmacological properties of cilostazol, a new antithrombotic drug. Part II: Effect on the peripheral organs. Arzneimittelforschung.

[24] Chen H, Ordög T, Chen J, Young DL, Bardsley MR, Redelman D, Ward SM, Sanders KM (2007). Differential gene expression in functional classes of interstitial cells of Cajal in murine small intestine. Physiol Genomics.

[25] Young HM (1999). Embryological origin of interstitial cells of Cajal. Microsc Res Tech.

[26] Vanderwinden JM, Rumessen JJ (1999). Interstitial cells of Cajal in human gut and gastrointestinal disease. Microsc Res Tech.

[27] West RB, Corless CL, Chen X, Rubin BP, Subramanian S, Montgomery K, Zhu S, Ball CA, Nielsen TO, Patel R, Goldblum JR, Brown PO, Heinrich MC (2004). The novel marker, DOG1, is expressed ubiquitously in gastrointestinal stromal tumors irrespective of KIT or PDGFRA mutation status. Am J Pathol.

[28] Miettinen M, Wang ZF, Lasota J (2009). DOG1 Antibody in the Differential Diagnosis of Gastrointestinal Stromal Tumors. Am J Surg Pathol.

[29] Wechsler J, Choi YH, Krall J, Ahmad F, Manganiello VC, Movsesian MA (2002). Isoforms of Cyclic Nucleotide Phosphodiesterase PDE3A in Cardiac Myocytes. J Biol Chem.

[30] Surapisitchat J, Jeon KI, Yan C, Beavo JA (2007). Differential Regulation of Endothelial Cell Permeability by cGMP via Phosphodiesterases 2 and 3. FASEB J.

[31] Hambleton R, Krall J, Tikishvili E, Honeggar M, Ahmad F, Manganiello VC, Movsesian MA (2005). Isoforms of cyclic nucleotide phosphodiesterase PDE3 and their contribution to cAMP hydrolytic activity in subcellular fractions of human myocardium. J Biol Chem.

[32] Bahlawane C, Schmitz M, Letellier E, Arumugam K, Nicot N, Nazarov P V, Haan S (2016). Data on quantification of signaling pathways activated by KIT and PDGFRA mutants. Data Br.

[33] Marko D, Romanakis K, Zankl H, Fürstenberger G, Steinbauer B, Eisenbrand G (1998). Induction of apoptosis by an inhibitor of cAMP-specific PDE in malignant murine carcinoma cells overexpressing PDE activity in comparison to their nonmalignant counterparts. Cell Biochem Biophys.

[34] McEwan DG, Brunton VG, Baillie GS, Leslie NR, Houslay MD, Frame MC (2007). Chemoresistant KM12C colon cancer cells are addicted to low cyclic AMP levels in a phosphodiesterase 4-regulated compartment via effects on phosphoinositide 3-kinase. Cancer Res.

[35] Shimizu K, Murata T, Watanabe Y, Sato C, Morita H, Tagawa T (2009). Characterization of phosphodiesterase 1 in human malignant melanoma cell lines. Anticancer Res.

[36] Murata K, Kameyama M, Fukui F, Ohigashi H, Hiratsuka M, Sasaki Y, Kabuto T, Mukai M, Mammoto T, Akedo H, Ishikawa O, Imaoka S (1999). Phosphodiesterase type III inhibitor, cilostazol, inhibits colon cancer cell motility. Clin Exp Metastasis.

[37] Schrör K (2002). The pharmacology of cilostazol. Diabetes, Obes Metab.

[38] Shaul YD, Yuan B, Thiru P, Nutter-Upham A, McCallum S, Lanzkron C, Bell GW, Sabatini DM (2016). MERAV: A tool for comparing gene expression across human tissues and cell types. Nucleic Acids Res.

[39] Seamon KB, Padgett W, Daly JW (1981). Forskolin: unique diterpene activator of adenylate cyclase in membranes and in intact cells. Proc Natl Acad Sci U S A.

[40] Aoki O, Hattori Y, Tomizawa A, Jojima T, Kasai K (2010). Anti-inflammatory role of cilostazol in vascular smooth muscle cells in vitro and in vivo. J Atheroscler Thromb.

[41] Jung WK, Lee DY, Park C, Choi YH, Choi I, Park SG, Seo SK, Lee SW, Yea SS, Ahn SC, Lee CM, Park WS, Ko JH (2010). Cilostazol is anti-inflammatory in BV2 microglial cells by inactivating nuclear factor-kappaB and inhibiting mitogen-activated protein kinases. Br J Pharmacol.

[42] Okutsu R, Yoshikawa T, Nagasawa M, Hirose Y, Takase H, Mitani K, Okada K, Miyakoda G, Yabuuchi Y (2009). Cilostazol inhibits modified low-density lipoprotein uptake and foam cell formation in mouse peritoneal macrophages. Atherosclerosis.

[43] Takaguri A, Morimoto M, Imai S ichi, Satoh K (2016). Cilostazol inhibits interleukin-1-induced ADAM17 expression through cAMP independent signaling in vascular smooth muscle cells. Cell Biol Int.

[44] Schindelin J, Arganda-Carreras I, Frise E, Kaynig V, Longair M, Pietzsch T, Preibisch S, Rueden C, Saalfeld S, Schmid B, Tinevez JY, White DJ, Hartenstein V (2012). Fiji: an open source platform for biological image analysis. Nat Methods.

[45] Preibisch S, Saalfeld S, Tomancak P (2009). Globally optimal stitching of tiled 3D microscopic image acquisitions. Bioinformatics.

[46] Chou TC (2006). Theoretical Basis, Experimental Design, and Computerized Simulation of Synergism and Antagonism in Drug Combination Studies. Pharmacol Rev.

