# Supplementary material for: Phosphodiesterase 3A: a new player in development of interstitial cells of Cajal and a prospective target in gastrointestinal stromal tumors (GIST)
Source: Oncotarget. 2017 Apr 10;8(25):41026–43. doi: 10.18632/oncotarget.17010 (PMC5522287; doi:10.18632/oncotarget.17010)
Supplement: Supplementary file 1 [file oncotarget-08-41026-s001.pdf]

Phosphodiesterase 3A: a new player in development of interstitial cells of Cajal and a prospective target in gastrointestinal stromal tumors (GIST)

Supplementary Materials

SUPPLEMENTARY FIGURES AND TABLES

```
humanPDE3A
           1076 TQHLLQNHKMWKKVIEEEQRLAGIENQSLDQTPQSHSS 1113

           1076 TQHLLQNHMMWKKVIEEEQCLSGTENQSLDQVPLQHPS 1113
mousePDE3A
```

Supplementary Figure 1: Alignment of the amino-acid sequence of the immunogenic peptide used to raise the anti-human PDE3Aantibody. Black box highlights the sequence of the human PDE3A immunogenic peptide [20], aligned to the mouse PDE3A peptide sequence (NCBI Reference Sequence: NP_000912.3 and NP_061249.1). Red indicates missmatched amino-acids. Green indicates matching amino-acids.

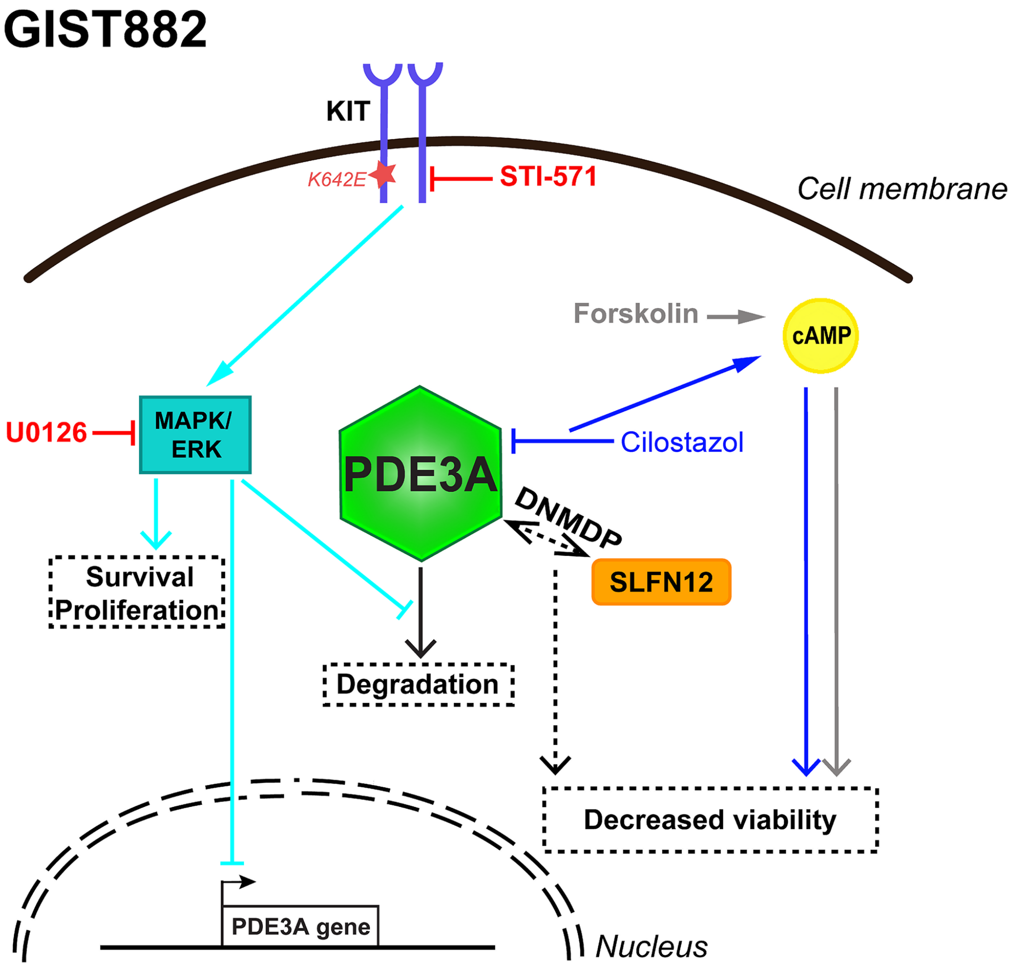

Supplementary Figure 2: Graphical model of the PDE3A interplay in GIST882 cells. Plain cyan arrows: Oncogenic mutation (K642E) of the KIT receptor leads to activation of MAPK/ERK pathway and promotes cell viability. Other signaling pathways downstream of KIT receptor not represented here for the sake of clarity. Plain red arrows: KIT inhibition by STI-571 downregulates the MAPK/ERK pathway, decreases the amount of PDE3A protein and simultaneously upregulates PDE3A mRNA. The MEK inhibitor U0126 replicates the effect of STI-571, suggesting a central role for the MAPK/ERK pathway in PDE3A protein and mRNA turn-over in GIST882 cells. Plain blue arrows: the PDE3 inhibitor cilostazol reduces GIST882 cell viability without affecting the MAPK/ ERK pathway (pERK). (not shown) cilostazol synergizes with STI-571 to reduce GIST882 cell viability (Chou- Talalay's combination index <1). Refer to results section for details. Plain grey arrows: Forskolin, a direct activator of adenylyl cyclase, reduced GIST882 viability and synergizes with cilostazol (Chou-Talalay's combination index <1). Refer to result section for details. Dashed black arrows: GIST882 cells express both PDE3A and SLFN12. DNMDP exerts a cytotoxic effect on GIST882 cells, likely by inducing a neomorphic protein-protein interaction between PDE3A and SLFN12, as recently described by de Waal *et al.* in other cancer cells [18].

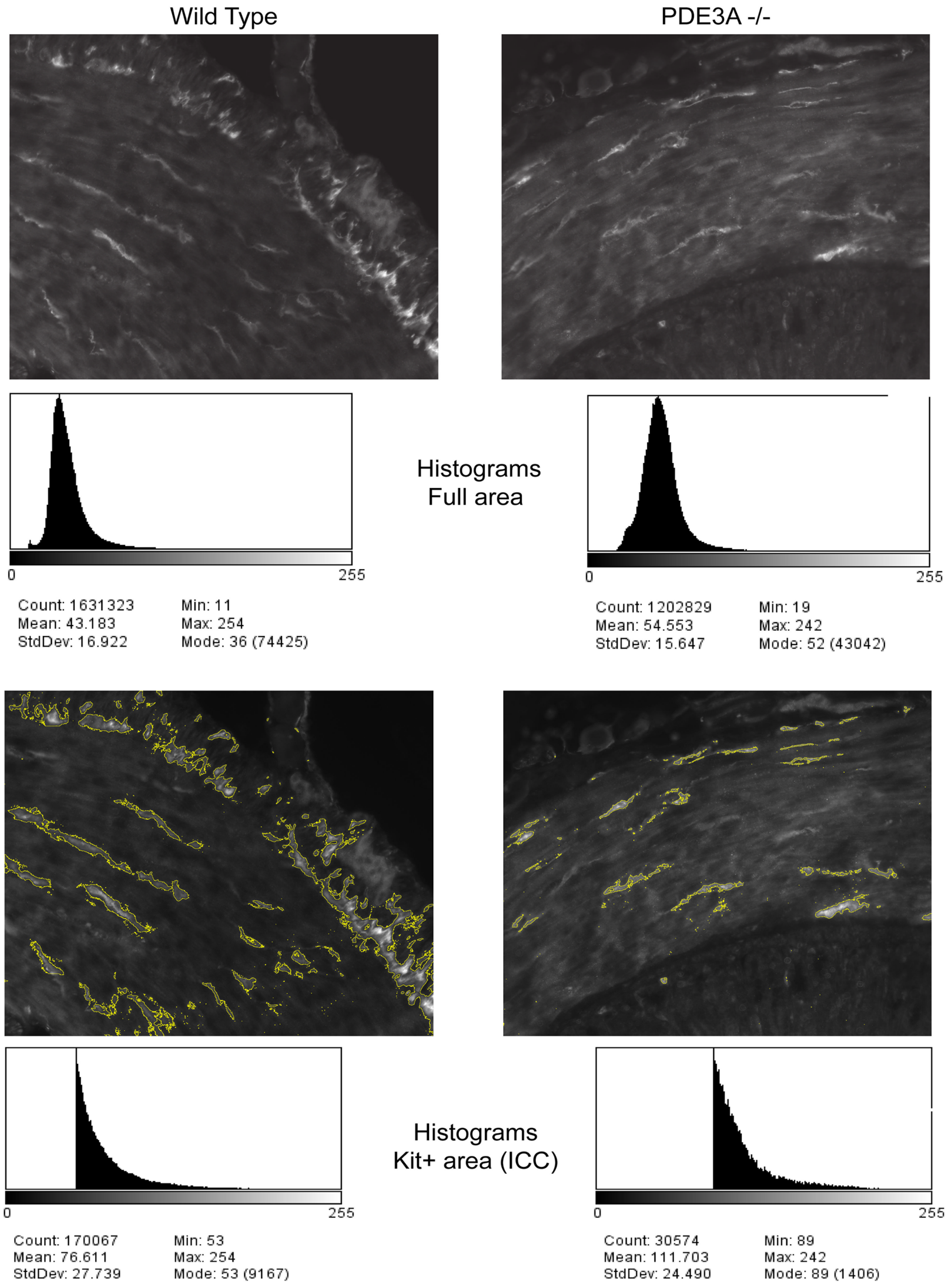

Supplementary Figure 3: KIT-ir intensity is similar in ICC of WT and PDE3A-/- mice. Upper panels: Representative fields of view of WT (left) and PDE3A-/- (right) muscularis propria stained with KIT antibody. Histograms of global pixel intensity values of both pictures are within the same range. Lower panels: Same fields of view with segmented KIT+ areas highlighted in yellow. Histogram of pixel intensity values for the segmented KIT+ area are within the same range. The reduction of KIT+ ICC observed in PDE3A -/- gut cannot be attributed to a reduced level of KIT-ir intensity. The similar distribution of KIT-ir intensity in WT and PDE3A-/- gut indicates a genuine reduction of the number of KIT+ ICC in the PDE3A -/- gut. Scale bar = 50µm.

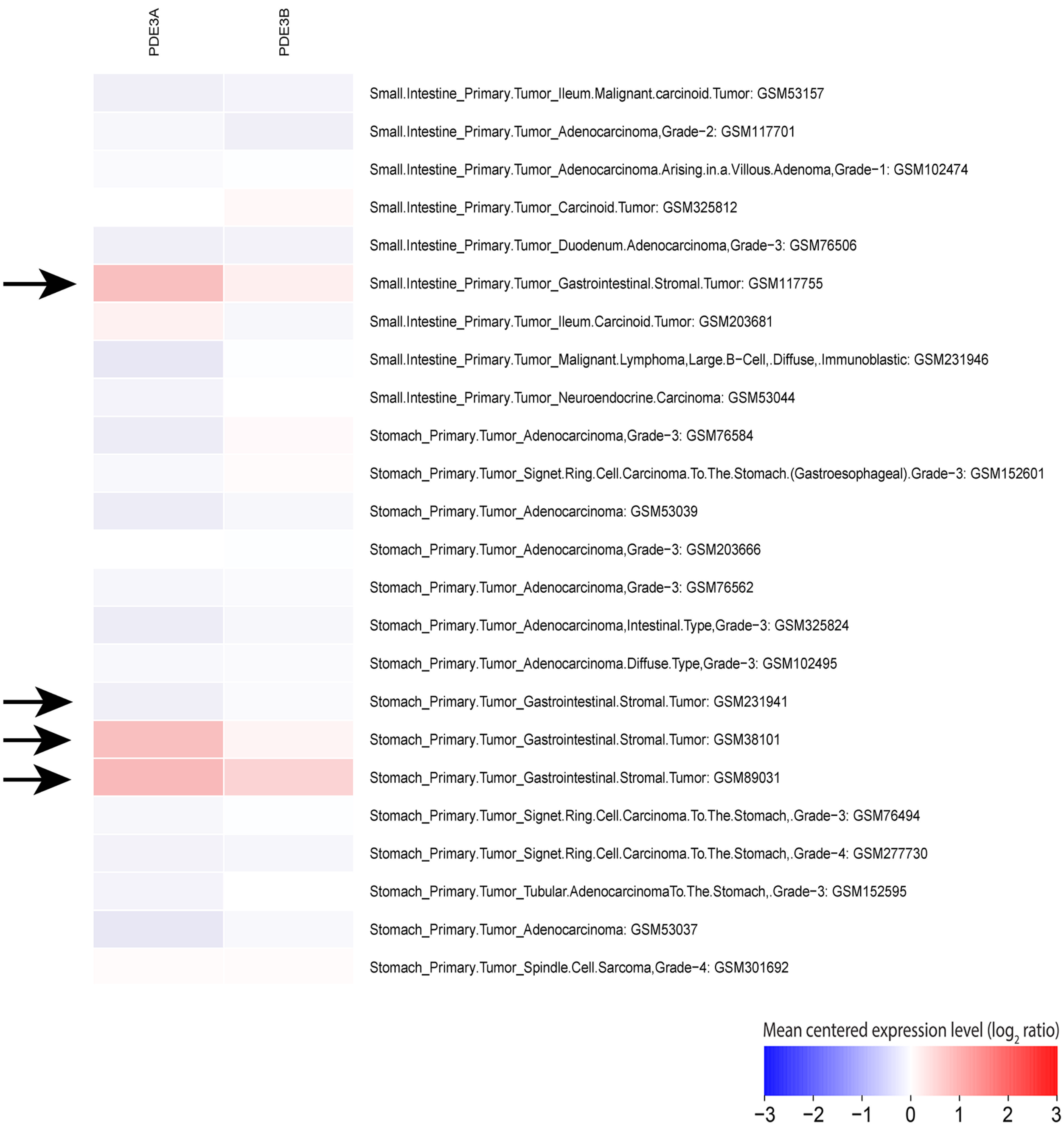

Supplementary Figure 4: PDE3A and PDE3B expression in primary gastrointestinal stromal tumors. Heatmap of PDE3A and PDE3B expression in primary tumors of digestive tract [36]. Black arrows indicate gastrointestinal tumors. PDE3A appears generally more expressed than PDE3B in primary GIST.

Supplementary Table 1: Primary and secondary antibodies for immunostaining

Primary antibodies	Supplier	Cat. N°	Host	Dilution
SLFN12	Abcam	ab113238	Rabbit	1/500
hPDE3A (1095-1110)	MRC-PPU Reagents	S721A	Sheep	1/2000
mPDE3A (1098-1115)	NHLBI [19]	N/A	Rabbit	1/500
alphaSMA	Sigma	C6198	Mouse	1/30.000
KIT (D13A2)	Cell signaling technology	3074	Rabbit	1/500
KIT	DAKO	A4502	Rabbit	1/500
**Secondary antibodies**	**Supplier**	**Cat.N°**	**Host**	**Dilution**
Anti sheep Biotin-SP	Jackson Immunoresearch laboratories, Inc.	713-065-147	Donkey	1/200
Anti rabbit Biotin-SP	Jackson Immunoresearch laboratories, Inc.	711-065-152	Donkey	1/200
Anti sheep Alexa 488	Jackson Immunoresearch laboratories, Inc.	713-545-147	Donkey	1/200
Anti rabbit Alexa 594	Jackson Immunoresearch laboratories, Inc.	711-585-152	Donkey	1/200
Anti rabbit Alexa 647	Jackson Immunoresearch laboratories, Inc.	711-605-152	Donkey	1/200

Supplementary Table 2: Primary and secondary antibodies for Western blot

Primary antibodies	Supplier	Cat. N°	Host	Dilution
SLFN12	Abcam	ab113238	Rabbit	1/200
hPDE3A (1095-1110)	MRC-PPU Reagents	S721A	Sheep	1/500
mPDE3A (1098-1115)	NHLBI [19]	N/A	Rabbit	1/200
ERK	Cell signaling technology	9102	Rabbit	1/500
pERK Thr202/Tyr204	Cell signaling technology	9106	Mouse	1/500
GAPDH (14C10)	Cell signaling technology	2118	Rabbit	1/200
**Secondary antibodies**	**Supplier**	**Cat.N°**	**Host**	**Dilution**
Anti sheep Alexa 680	Jackson Immunoresearch laboratories, Inc.	713-625-147	Donkey	1/10.000
Anti rabbit 680RD	LI-COR	925-68073	Donkey	1/10.000
Anti mouse 800CW	LI-COR	925-32212	Donkey	1/10.000

Supplementary Table 3: Primers used on human GIST882 cells

Primers	Sequence
GAPDH Fw	TGTGTCCGTCGTGGATCTGA
GAPDH Rev	CCTGCTTCACCACCTTCTTGA
β-Actin Fw	AACCGTGAAAAGATGACCCAGAT
β-Actin Rev	GCCTGGATGGCTACGTACATG
PDE3A Fw	TTTCCTTAGAGAGGTTCAAGGTCG
PDE3A Rev	AATACTGGTTCCTGAAGACTGTGAT

Supplementary Table 4: Clinicopathologic features of SuperBiochips GIST TMA

**SuperBiochips GIST TMA**	
**Sex/Age average**	**Total (n)/years**
Male	28/58.3
Female	22/62.6
**Primary tumor site**	**Total (n)**
Gastric	24
Small bowel	15
Abdominal cavity	1
Rectum	2
Disseminated	8
**Tumor morphology**	**Total (n)**
Spindle	47
Epithelioid	3
**Risk category**	**Total (n)**
Malignant	8
High risk	26
Intermediate risk	8
Low risk	8
**Mitotic Figures**	**Total (n)**
≤5/50	24
>5/50	26
**KIT-ir**	**Total (n)**
positive	50
negative	0

Supplementary Table 5: Clinicopathologic features of CMMI DiaPath GIST TMA

CMMI DiaPath GIST TMA	
**Sex/Age average**	**Total (n)/years**
Male	34/58.3
Female	22/62.6
**Primary tumor site**	**Total (n)**
Gastric	26
Small bowel	22
Colon	1
Disseminated	5
**Tumor morphology**	**Total (n)**
Spindle	64
Epithelioid	11
**Risk category**	**Total (n)**
Malignant	14
High risk	12
Intermediate risk	9
Low risk	21
**Mitotic Figures**	**Total (n)**
≤5/50	32
>5/50	24
**KIT-ir**	**Total (n)**
positive	67
negative	8

Supplementary Table 6: Biothèque de Liège, FFPE GIST slides clinicopathologic features

Code	Sex	Age	Organ	Diagnostic	Type	Immunostaining +	Immunostaining -
BPGIST10/05	M	62	Duodenum	Malignant GIST	Primary	Vimentin<br>CD117	Desmin<br>Neurofilaments<br>Actin<br>S100<br>Ki64<br>CD34
BPGIST10/09	F	66	Stomach	GIST	Primary	Vimentin<br>CD34<br>CD99	Factor VIII<br>CD117<br>EMA<br>NSE<br>S100
BPGIST10/11	M	68	Stomach	GIST	Primary	Vimentin<br>CD34<br>CD117	Actin<br>Desmin<br>Actine<br>S100<br>SMMHC
BPGIST10/17	F	65	Stomach	Malignant GIST	Primary	CD117<br>CD34<br>Desmin<br>S100<br>Ki-67 (5-10%)	Actin
